# Study of the Relationship between ICU Patient Recovery and TCM Treatment in Acute Phase: A Retrospective Study Based on Python Data Mining Technology

**DOI:** 10.1155/2021/5548157

**Published:** 2021-03-02

**Authors:** Zhiqun Wu, Xue Wang, Renlong Pan, Xiufu Huang, Yuhan Li

**Affiliations:** ^1^Department of Neurosurgery, Shanghai Blue Cross Brain Hospital, Qixin Road 2880, Shanghai 201100, China; ^2^Intensive Care Unit (ICU), Shanghai Tianyou Hospital, Zhen'nan Road 528, Shanghai 200333, China

## Abstract

**Background:**

Data was mined with the help of an artificial intelligence system based on Python, data was collected, and a database was established using a Python crawler, and the relationship between the outcome of neurosurgery ICU patients and treatment using traditional Chinese medicine was ascertained through data management and statistical processing.

**Method:**

The source data cases (*n* = 2237) were selected. By following the experimental design, data (*n* = 739) were obtained through artificial intelligence processing, including *n* = 480 in the group with traditional Chinese medicine treatment and *n* = 259 in the group without traditional Chinese medicine treatment. An evaluation was carried out using characteristics of patents' ICU stays and summated rating scales.

**Results:**

There were statistical differences in 5 evaluation items (*P* < 0.05), and other comparison items also showed data with results favoring the outcomes in the intervention group using traditional Chinese medicine. *Discussion*. Traditional Chinese medicine as an alternative medical protocol effectively alleviates the stress and treatment fatigue brought about by modern medicine. Artificial intelligence data mining is a favorable medium to quantify this. Python will play a greater role in future clinical research because of its own characteristics.

## 1. Background

In the treatment of neurosurgery ICU patients, in addition to conventional ICU medication, nursing, and monitoring, traditional Chinese medicine has gradually become an alternative medical protocol that cannot be ignored [1]. However, the clinical evidence for the application of Chinese medicine in the treatment of seriously ill patients, especially in neurosurgery ICU patients, is insufficient. However, methods using Chinese medicine are still welcomed by patients and their families, as well as by ICU medical staff [2]. Is TCM really effective or are we just seeing the “survivorship bias”? In this paper, the author attempts to explore the role of traditional Chinese medicine in the treatment of neurosurgical ICU patients by looking at clinical research evidence [3, 4].

Data mining technology is a kind of data processing technology. It is a process of extracting potentially useful information and knowledge of which we are not aware beforehand from a large amount of incomplete, noisy, fuzzy, and random data. For data mining, the appropriate analysis tools need to be selected based on the data information in the data warehouse [5], and the information needs to be processed using statistical methods, case-based reasoning, decision trees, rule-based reasoning, fuzzy sets, even neural networks, and genetic algorithms to obtain useful information for analysis. The process of data mining is a “recycling” process. If at any step the expected goal is not achieved, we need to go back to the previous steps, readjust them, and execute them. Data mining technology has been applied relatively early to clinical research, with a relatively wide range of applications [6]. However, in this research, the current relatively advanced high-level computer language “Python” was adopted to design the data mining program code. Python is a high-level scripting language that combines interpretation, compilation, interactivity, and object orientation. Python was designed to have a strong readability. Compared with other languages, it often uses English keywords and some punctuation marks from other languages. In addition, it has more distinctive grammatical structures than other languages. Python has many advantages, such as its ease of learning, readability, ease of maintenance, extensive standard library, interactive mode, portability, extensibility, database [7], GUI programming, and embeddability. The prime advantage is that it is powerful, friendly to beginners, and naturally attractive to clinical workers [8].

Data was collected and a database was established using a Python crawler. Afterwards, code was written with Python [9]. A large number of clinical data were mined [10]. The relationship between neurosurgery ICU patients and traditional Chinese medicine treatment was ascertained through statistical processing [11].

## 2. Methods

### 2.1. Data Selection

**Figure 1:**
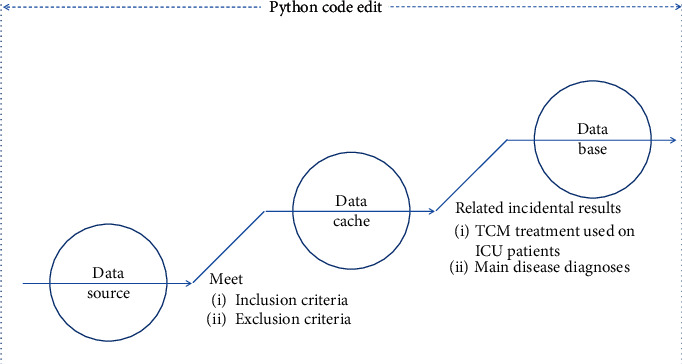
Data mining schematic.

From June 2015 to October 2020, all electronically stored cases (*n* = 2237) of patients who had been admitted to the ICU at the Neurosurgery Department of Blue Cross Brain Hospital affiliated with Shanghai Tongji University were selected. The research database was established by using Python data mining technology [12]. The STROBE checklist was used as the experiment design guidance method, and the data were cleaned according to the inclusion and exclusion criteria [13]. The preprocessed data (*n* = 739) were obtained. The preprocessed data were divided into two parts according to the actual clinical treatment methods used in the cases: a group with traditional Chinese medicine treatment (*n* = 480) and a group without traditional Chinese medicine treatment (*n* = 259). For the data mining schematic, please see Figure 1.

#### 2.1.1. Inclusion Criteria

Inclusion criteria are as follows [14]: (a) cases with a clear ICD-10 diagnosis code; (b) hospital admission cases during the target time period; (c) cases that have complete medical records and can match the evaluation requirements; (d) cases with a history of transfer to a neurosurgery department ICU; (e) cases whose data can be crawled by a Python crawler.

It is particularly necessary to explain that in this research, when beginning to use Python to write the code, the private information in the patients' medical records, such as name, date of birth, residential address, and other information that cannot be crawled by a Python crawler, was prevented from being captured at the data mining tool level. Therefore, ethical issues in this scientific research were effectively avoided. The ethical committee demonstrated that informed consent cannot be signed because there is no clear subject in this research.

#### 2.1.2. Exclusion Criteria

Exclusion criteria are as follows: (a) patients with an unclear diagnosis, or with secondary or primary serious cardiovascular or cerebrovascular diseases, or with liver, kidney, hematopoietic system, or other diseases; (b) patients with mental illness and in a stage of acute onset; (c) patients with incomplete medical records or a lack of important information regarding diagnosis and treatment; and (d) patients whose case records are not compatible with the STROBE checklist.

### 2.2. Interventions

#### 2.2.1. The Group with Traditional Chinese Medicine Treatment

**Figure 2:**
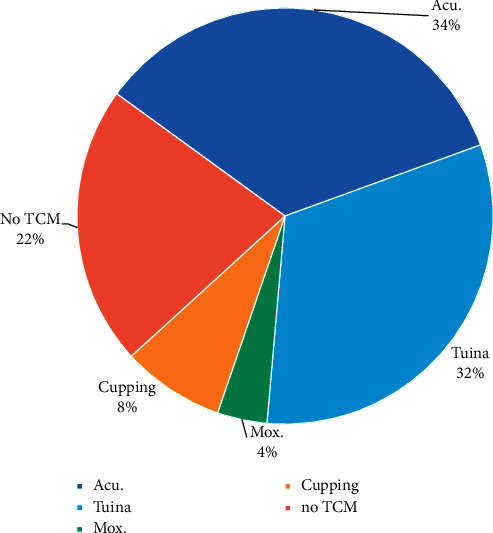
Several forms of TCM that were used in treatment. A histogram or pie chart of the four methods: acupuncture, cupping, Tuina, and moxibustion.

The subjects included in this group received a clear traditional Chinese medicine treatment intervention in the treatment process. After being processed by Python artificial intelligence, the data showed that these interventions were mainly acupuncture, cupping, massage, and moxibustion. Please see Figure 2.

#### 2.2.2. The Group without Traditional Chinese Medicine Treatment

The subjects included in this group did not receive treatment from traditional Chinese medicine during treatment. Only conventional ICU treatment protocols were used.

#### 2.2.3. Python Code and Workflow

**Figure 3:**
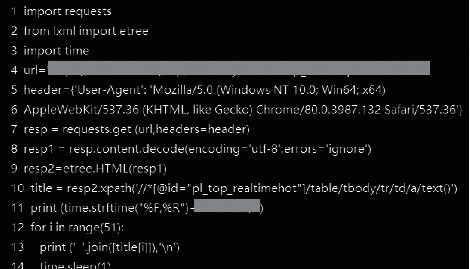
Some Python code screenshots.

(a) For data acquisition, organize the database, write the crawler code, and use the Python crawler to obtain the basic database material [15]. (b) For data management, through the data mining process, starting from data integration, through data cleaning, data selection, data collation, and other processes, obtain the preprocessed data. (c) Divide the preprocessed data into groups according to the experimental design for statistical comparison. Please see Figure 3.

## 3. Evaluation

In addition to the regular evaluation methods, that is, ICU days, urinary catheter removal time, nasal feeding tube removal time, ventilator weaning time, and the time to first flatus after surgery, evaluation methods such as the APACHE II Score (Acute Physiology and Chronic Health Score II) [16], GCS Score (Glasgow Coma Score), Delirium Assessment Scale (CAM-ICU), and Murray Lung Injury Score were selected and adopted.

### 3.1. The Apache II Score (Acute Physiology and Chronic Health Score II) System

The APACHE II score is composed of acute physiology scores, the age score, and chronic health scores. The higher the score, the more severe the disease, the worse the prognosis, and the higher the mortality rate. The Acute Physiology Score (APS) includes 12 physiological indices. The worst values (the highest or the lowest values) in the first 24 hours of ICU admission are selected and scored according to the attached table, and the higher scores are selected. The age score is divided into 5 stages from below 44 years old to above 75 years old, respectively, rated as A6 points. For the chronic health score, patients are required to meet the diagnosis of chronic organ dysfunction or immunosuppression before admission. For patients with chronic organ dysfunction or in a state of immunosuppression, if they are admitted to ICU after elective surgery, the score is 2 points, and for those who are admitted to ICU after emergency surgery or nonsurgery, the score is 5 points. The final APACHE II score is the sum of the three scores. The APACHE II score was originally designed with the worst score within 24 hours of entering the ICU, so it is generally not used for the continuous dynamic evaluation of the severity of patients' condition.

### 3.2. The GCS Score (Glasgow Coma Score)

Evaluation using the Glasgow Coma Index includes three aspects: eye opening response, language response, and body movement. The sum of these three points is the coma index. The coma index is a medical index to evaluate the degree of coma degree in patients. Nowadays, the Glasgow Coma Index is the most widely used index.

### 3.3. Delirium Assessment Scale (CAM-ICU)

The diagnosis of delirium is mainly based on clinical examination and history. The confusion assessment method for the diagnosis of delirium in the ICU mainly includes the following aspects: the patient shows sudden changes or fluctuations in their state of consciousness, a lack of concentration, or confusion of thought and decreased clarity of consciousness.

### 3.4. Murray Lung Injury Score

Murray proposed a scoring method for the degree of lung injury in 1988. This scoring method makes a quantitative analysis of the degree of lung injury in acute respiratory distress syndrome (ARDS). Murray evaluated the degree of lung injury according to the partial pressure of oxygen in arterial blood/fractional inspired oxygen (PaO2/FiO2), the level of positive end-expiratory pressure (PEEP), the scores of the number of affected quadrants in chest X-rays, and the changes in lung compliance. The patient is evaluated as having acute lung injury, that is, ARDS, if the score is greater than 2.5. Patients with scores of 0.1–2.5 are evaluated a having mild-to-moderate lung injury. This standard emphasizes the continuous developmental process of lung injury from mild to severe and provides quantitative evaluation for lung injury. Studies by Owens et al. showed that the lung injury score had a significantly positive correlation with the extent of affected lung tissue (*r* = 0.75, *P* < 0.01) and was also closely associated with pulmonary vascular permeability (*r* = 0.73, *P* < 0.01). It can be seen that the Murray Lung Injury Score can accurately evaluate the degree of lung injury and is currently the most widely used score in clinical studies.

## 4. Statistical Analyse

SPSS 19.0 statistical software was adopted to conduct a statistical analysis of the data. Measurement data were expressed by mean and standard deviation. A paired *t*-test was adopted for comparison within groups, and an independent sample *t*-test was adopted for comparison among groups. Count data were expressed by rate or constituent ratio, and a chi-square test was adopted for comparison among groups. A Ridit analysis was adopted for comparison among groups of ranked data. *P* < 0.05 indicates that the difference is statistically significant.

## 5. Flow Chart

**Figure 4:**
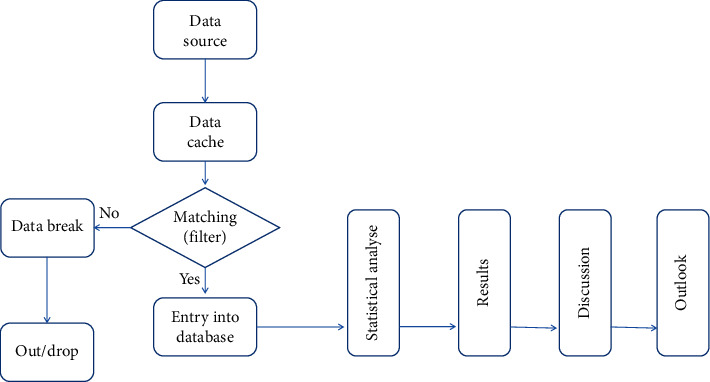
Flow chart for the study from data source mining to the end. The date was collected from the source into data cache, with the filter working following the inclusion/exclusion criteria; date was divided into two parts, only when the data matched; it can be put into database. Otherwise, the other part of data will be broken down and taken out of the study.

Flow chart of the study is shown in Figure 4.

## 6. Results

Through mining and processing by the artificial intelligence program, 739 cases conforming to data requirements were found and included, including 480 cases from the group with traditional Chinese medicine treatment and 259 cases from the group without Chinese medicine treatment. The main diseases covered included neurosurgical diagnoses such as cerebral hemorrhage (ICD-10 I61.902), craniocerebral trauma (ICD-10 S06.9059), subarachnoid hemorrhage (ICD-10 I60.901), and aneurysm rupture (ICD-10 I60.801).

### 6.1. Baseline

The baseline data is listed in Table 1.

### 6.2. Data Comparison between ICU Patients with and without TCM Treatment

The comparison data between ICU patients with and without TCM treatment are shown in Table 2.

The data mined through Python artificial intelligence are used for conducting statistical processing with the data obtained after processing. As mentioned in Tables 1 and 2 above, among brain surgery ICU patients treated with TCM, in each comparison item such as ICU hospital stay, urinary catheter removal time, nasal feeding tube removal time, ventilator weaning time, and time to first flatus after surgery, the durations were shorter than those in the group without TCM. Among these, the *P* value of the ICU hospital stay, the urinary catheter removal time, time to first flatus after surgery, and the time to first improved muscle strength was less than 0.05, which shows a statistically significant difference. For the evaluation of muscle strength at discharge, according to the GCS score at discharge, APACHE II (Acute Physiology and Chronic Health Score II) scoring system at discharge, VAS score at discharge, and Delirium Evaluation Scale at discharge (CAM-ICU), the group with TCM treatment performed better than the group without TCM treatment. However, only the GCS scoring items, *P* < 0.05, showed a statistically significant difference. In the evaluations according to the number of cases of ICU-acquired delirium and the number of cases of ICU-acquired weakness, the values in the group with TCM treatment were less than the values in the group without TCM treatment; however, there was no statistically significant difference.

## 7. Discussion and Outlook

TCM and Western medicine study the same issue, that is, the health and illness of people. The main issue lies in the fact that their perspectives are different. Therefore, the two have their own characteristics and advantages and also have their limitations. If they can bring their respective advantages together, then the best diagnosis and treatment protocols can be provided to patients [17]. In a difficult surgery, the success on the operating table is only half a success. The ICU is the guarantee of a successful surgery, having a high requirement for equipment, environment, and talent [18]. However, the ICU is limited by a higher level of work intensity needed and greater pressure on the patient, and it often leads to excessive fatigue. This is also reflected in the results of patient treatment. The alternative medical protocol of introducing TCM has effectively alleviated the pressure caused by modern healthcare, intensive healthcare, intensive equipment use, and intensive treatment [18]. The above results show the impressive effect that TCM has on the treatment of patients in the neurosurgery ICU and provide an ideal improved treatment solution in the neurosurgery ICU. Due to the benefits of data mining technology, this fact can be presented to us by using the data.

With data mining technology, we can find potential and meaningful patterns and associations from the massive amount of information in the real world, especially in the context of the current fierce collision between modern medicine and traditional medicine. With deeper research, more and more investigators are using data (such as frequency statistics, association rules, and cluster analyses) to explore potential relationships between modern medicine and traditional medicine. Various data mining technologies are being applied to the model of each department of traditional medicine and clinical medicine and are showing a relatively high accuracy. However, there is currently a lack of verification of the effectiveness of such models and of comparative analysis with traditional disease evaluation tools. In the future, it will be necessary to carry out multicenter and prospective studies to verify the effectiveness of these models. There is a certain conflict between the professionalism of clinical doctors and the development of data mining technology in medical data. In comparison with numerous data mining tools, Python distinguishes itself due to its ease of learning and use. Additionally, Python meets the requirements of artificial intelligence because of its powerful functionality. At this stage, after decades of development, Python has entered the stage of Python 3.1 and above, and an increasing amount of medical workers is beginning to use it to conduct scientific study designs and research applications relating to advanced artificial intelligence.

However, there are still areas that need to be further perfected in the future. For example, in this research, we only discussed the difference between the outcomes of patients treated with TCM and without TCM; however, we did not conduct a data-based exploration on whether there is a difference in the curative effect according to specific methods of TCM. There were 4 main diseases involved in this research, but there was, for example, no discussion carried out on the basis of each disease as a subgroup. The reason for this is that there are still relatively few reports from retrospective studies of clinical data using Python, a complete exploration system has not yet been formed, and there is still space for development regarding the benefits of using Python for writing data mining artificial intelligence systems. It is believed that more clinical scientific study protocols based on this computer language will emerge in the future and will become an important part of clinical scientific study and data management with respect to artificial intelligence deep learning and neural networks.

## Figures and Tables

**Table 1:** Baseline data.

Baseline data		
	With TCM	Without TCM
The final number of cases included in the research	*N* = 480	*N* = 259
Age (mean ± SD)	67.38 ± 25.62	69.21 ± 28.10
Gender (male/female) (*n* case)	209/271	102/157
Maximum weight (kg)	126	110
Minimum weight (kg)	38	42
BMI (mean ± SD)	22.17 ± 10.66	23.41 ± 11.09
Cerebral hemorrhage (n cases)	162	97
Cerebral hemorrhage death (*n* cases)	29	8
Craniocerebral trauma (*n* cases)	140	82
Craniocerebral trauma death (*n* cases)	11	6
Subarachnoid hemorrhage (*n* cases)	62	35
Subarachnoid hemorrhage 3 death (*n* cases)	8	3

**Table 2:** Comparison data.

Data comparison between ICU patients with and without TCM treatment		
Comparison items (mean ± SD)	With TCM	Without TCM
The final number of cases included in the research	*N* = 480	*N* = 259
ICU hospital stay (days)	17.29 ± 11.03	22.91 ± 16.30
Urinary catheter removal time (days)	5.02 ± 4.73	7.74 ± 5.00
Nasal feeding tube removal time (days)	9.38 ± 6.27	11.60 ± 7.39
Ventilator weaning time (days)	12.49 ± 7.90	12.41 ± 8.51
Time to first flatus after surgery (days)	1.02 ± 1.33	1.97 ± 1.58
Time to first improved muscle strength (days)	2.37 ± 0.48	2.29 ± 0.99
Evaluation of muscle strength at discharge (grade)	3.80 ± 1.95	3.05 ± 2.06
VAS score at discharge (score)	2.75 ± 2.08	3.09 ± 1.55
Number of cases of ICU-acquired delirium (n)	42	32
Number of cases of ICU-acquired weakness (n)	30	15

## Data Availability

This research was registered (internally) at the Clinical Trial Registration Center at Shanghai Tongji University; all experimental data and related codes shall be saved here for 10 years. The original data, database and python code used to support the findings of this study are available from the corresponding author upon request.
